# Oleanolic Acid Alleviates Atopic Dermatitis-like Responses In Vivo and In Vitro

**DOI:** 10.3390/ijms222112000

**Published:** 2021-11-05

**Authors:** Yun-Mi Kang, Hye-Min Kim, Minho Lee, Hyo-Jin An

**Affiliations:** 1 Department of Pharmacology, College of Korean Medicine, Sangji University, Wonju 26339, Gangwon-do, Korea; yunmi6115@naver.com (Y.-M.K.); mins7576@daum.net (H.-M.K.); 2 Department of Life Science, Dongguk University-Seoul, Ilsandong-gu, Goyang-si 10326, Gyeonggi-do, Korea

**Keywords:** oleanolic acid, atopic dermatitis, 2,4-dinitrochlorobenzene, keratinocyte, NF-κB, STAT1

## Abstract

Oleanolic acid (OA) is a pentacyclic triterpenoid, abundantly found in plants of the *Oleaceae* family, and is well known for its beneficial pharmacological activities. Previously, we reported the inhibitory effect of OA on mast cell-mediated allergic inflammation. In this study, we investigated the effects of OA on atopic dermatitis (AD)-like skin lesions and its underlying mechanism of action. We evaluated the inhibitory effect of OA on AD-like responses and the possible mechanisms using a 1-chloro-2,4-dinitrochlorobenzene (DNCB)-induced AD animal model and tumor necrosis factor (TNF)-α/interferon (IFN)-γ-stimulated HaCaT keratinocytes. We found that OA has anti-atopic effects, including histological alterations, on DNCB-induced AD-like lesions in mice. Moreover, it suppressed the expression of Th2 type cytokines and chemokines in the AD mouse model and TNF-α/IFN-γ-induced HaCaT keratinocytes by blocking the activation of serine-threonine kinase Akt, nuclear factor-κB, and the signal transducer and activator of transcription 1. The results demonstrate that OA inhibits AD-like symptoms and regulates the inflammatory mediators; therefore, it may be used as an effective and attractive therapeutic agent for allergic disorders, such as AD. Moreover, the findings of this study provide novel insights into the potential pharmacological targets of OA for treating AD.

## 1. Introduction

Allergic inflammation is characterized by pathophysiological or hypersensitivity disorders, including allergic asthma, allergic rhinitis, anaphylaxis, and atopic dermatitis (AD), after exposure to allergens [1]. AD is a chronic inflammatory skin disease that arises from the complicated interaction of innate and adaptive immune responses based on genetics, environmental factors, immune abnormalities, and skin barrier functions [2]. The characteristic features of AD include itchy, swollen, red, and cracked skin with inflammatory cell accumulation in AD skin lesions. Although the pathogenesis of AD is not clear, it is known that several cells and factors are associated with its development. The pathological processes of AD are thought to be mediated by Th1/ Th2 balance, which is skewed toward Th2 in AD. Th2 cells are mainly activated in the acute phase of AD, while Th1 cells mediate the alteration of expression in chronic AD [3]. The standard treatment for AD involves the application of topical corticosteroids or the administration of immunosuppressive agents; however, protracted use of these agents can cause various side effects, such as skin atrophy, bleeding, vasodilation, and organ toxicity. For this reason, medicines originating from herbal sources may be preferred to steroids, and may be used in combination with other methods, such as enhancing immunity, reducing house mite dust, and dietary restrictions [4,5].

Numerous intracellular signal transduction triggered by ligand–cell surface receptor binding is mediated by transcription factors. Nuclear factor (NF)-κB and the signal transducer and activator of transcription (STAT)−1 are pivotal transcription factors associated with the allergic inflammatory response [6]. Upon stimulation, the inhibitor κB (IκB)-α protein is phosphorylated, leading to the ubiquitination and proteasomal degradation of IκB. Sequentially activated NF-κB and interferon (IFN)-γ-activated STAT 1 in the cytoplasm translocate into the nucleus, where they engage in the expression of numerous pro-inflammatory mediators. Thus, these transcription factors are important pharmacological targets for the discovery of novel therapeutics to treat allergic disorders [7,8].

Oleanolic acid (OA) is a pentacyclic triterpenoid that is abundant in plants of the *Oleaceae* family, such as *Olea europaea*. OA is ubiquitously found in food and plants, where it exists as a free acid or as an aglycone of triterpenoid saponins, such as ursolic acid, moronic acid, and betulinic acid [9]. To date, various reports have described the pharmacological activities of OA, including its antioxidant [10], anti-inflammatory [11,12], anti-asthmatic [13], anti-diabetic [14,15], anti-tumor [16], hepatoprotective [17], immunomodulatory [18], anti-parasitic [19], and anti-hypertensive [20] properties. Despite the fact that OA is a well-known active component contained in various plants, studies on its effect on AD are insufficient. As it is important to study natural materials that are effective against allergic diseases, we focused on OA that exhibits a wide range of biological activities, such as anti-inflammatory and anti-asthmatic effects, as a feasible active compound for allergic diseases. Previously, we reported the anti-allergic effect of OA, demonstrating that OA exerted an inhibitory effect on mast cell-mediated allergic inflammation in vivo and in vitro [21]. Allergic response and inflammation can trigger AD and worsen the condition, thus, controlling allergic and inflammatory reaction could be important strategy in the manage of AD. These results prompted us to investigate its potential effect on other allergic diseases, such as AD. As AD is mainly the beginning of a series of allergic disorders, we hypothesized that OA would attenuate AD-like symptoms. Thus, in the present study, we aimed to elucidate the effects of OA on AD-like lesions and define the underlying mechanisms of action using DNCB-induced AD mouse models and human keratinocytes.

## 2. Results

### 2.1. OA Attenuated AD Lesions in DNCB-Induced AD Mice

**Figure 1:**
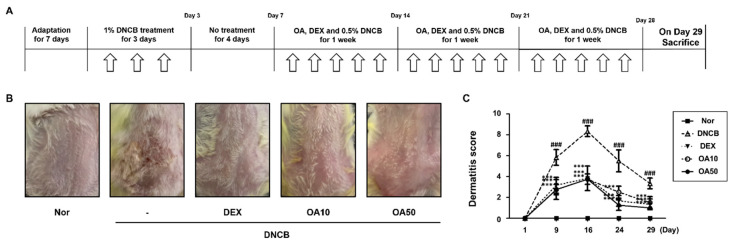
Effects of OA on DNCB-induced AD skin lesions in ICR mice. (**A**) Experimental schedule for the induction of AD. (**B**) Effect of OA on clinical features of DNCB-induced AD skin lesions. White arrows indicated DNCB treatment. (**C**) Effects of OA on dermatitis score. Densitometric analysis was performed using Bio-Rad Quantity One^®^ Software. The data shown represent mean ± S.D. (*n* = 6) of three independent experiments. ^###^ *p* < 0.001 vs. the control group; *** *p* < 0.001 vs. DNCB-treated group.

The repeated topical application of DNCB on the dorsal skin of mice induces AD skin symptoms. DNCB is a “contact sensitizer” that induces contact hypersensitivity of the skin in mice, which is considered to be a cell-mediated response [22]. To investigate the remedial effects of OA on AD mice, we administered OA following the induction of AD mouse skin. The experimental procedure is summarized in Figure 1A. On the day of sacrifice, severe AD-like lesions, such as erythema, edema, hemorrhage, scarring, dryness, excoriation, and erosion were observed on the dorsal skin of DNCB-induced AD mice. However, topical application of dexamethasone (10 μM), a well-known therapeutic agent for AD, and OA (10 and 50 μM) for 3 weeks significantly alleviated these AD skin symptoms compared to the DNCB group (*p* < 0.001) (Figure 1B,C).

### 2.2. OA Improved the Histological Observations and Histamine Release in DNCB-Induced AD Mice

**Figure 2:**
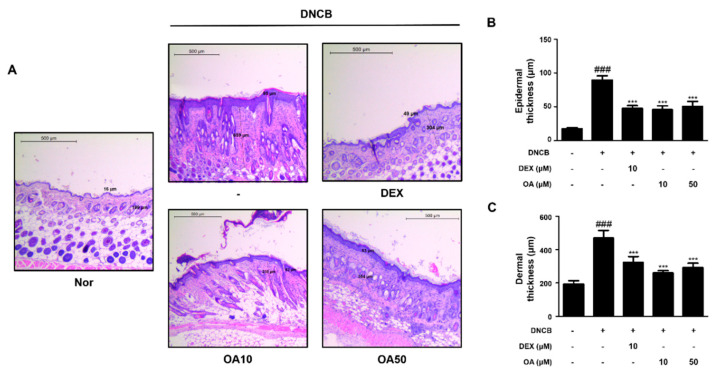
Effect of OA on epidermal and dermal thickness in DNCB-induced AD skin lesions. (**A**) H&E stained AD mouse skin lesions (scale bar = 500 μm). (**B**) Determination of epidermal thickness and (**C**) dermal thickness. Epidermal and dermal thickness in H&E stained sections were measured under a microscope. The data shown represent mean ± S.D. (*n* = 6) of three independent experiments. ^###^ *p* < 0.001 vs. the control group; *** *p* < 0.001 vs. DNCB-treated group.

**Figure 3:**
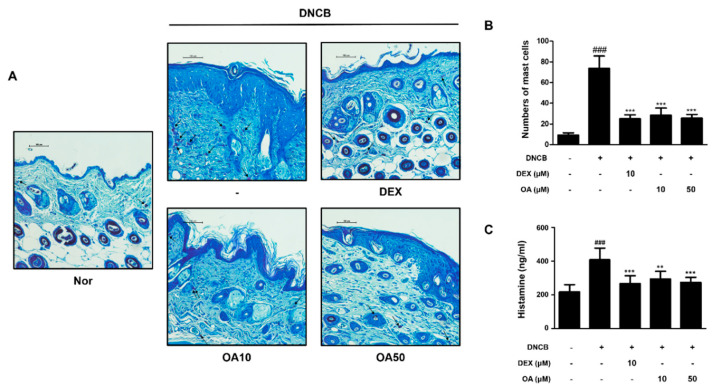
Effect of OA on mast cell infiltration and serum histamine level in DNCB-induced AD skin lesions. (**A**) Toluidine blue stained AD mouse skin lesions (scale bar = 100 μm). Black arrows indicated stained mast cells. (**B**) Number of mast cells per mm section. Mast cell infiltration in toluidine blue stained sections is expressed as the average total count in five fields. (**C**) Histamine release in mouse serum was measured using an ELISA kit. The data shown represent mean ± S.D. (*n* = 6) of three independent experiments. ^###^ *p* < 0.001 vs. the control group; ** *p* < 0.01 and *** *p* < 0.001 vs. DNCB-treated group.

Histological alterations, such as epidermal hyperplasia and infiltration of lymphocytes and mast cells in the skin, are the main hallmarks of AD [23]. Improvements in clinical skin conditions following OA treatment were confirmed by histological examination. Histological analysis was performed on atopic skin tissues. The excised skin from each group was stained with hematoxylin and eosin (H&E) or toluidine blue, and histological alterations were observed microscopically. H&E-stained tissue sections revealed that the thickness of epidermal and dermal tissues was greater in the DNCB-treated group (91.84 ± 7.60, 474.66 ± 43.65 μm, respectively, *p* < 0.001) than the control group due to edema, hyperkeratosis, and hyperplasia (Figure 2A). However, treatment with 10 and 50 μM OA markedly attenuated the epidermal (47.16 ± 5.98 and 52.65 ± 9.56 μm, *p* < 0.001) and dermal thickening (258.65 ± 17.56 and 292.65 ± 25.61 μm, *p* < 0.001) (Figure 2B,C). In the toluidine blue-stained tissue sections, mast cell infiltration, an indicator of inflammation, was noticeably increased in the DNCB-treated group compared to the control group (73.67 ± 12.06 cells, *p* < 0.001). Treatment with 10 and 50 μM OA attenuated the infiltration of inflammatory cells, particularly mast cells, as evidenced by toluidine blue staining (28.5 ± 6.98, 25.5 ± 3.73 cells, respectively, *p* < 0.001) (Figure 3A,B). As mast cells are sources of histamine, which is the most potent mediator involved in AD symptoms [24], histamine levels in the serum were also examined. The results showed that treatment with 50 μM OA remarkably inhibited histamine release (271.91 ± 35.75 ng/mL, *p* < 0.001) compared to the DNCB-treated group (411.81 ± 60.12 ng/mL, *p* < 0.001) (Figure 3C).

### 2.3. OA Suppressed the mRNA Expression of AD-Related Cytokines and Activation of IκB and STAT1 in DNCB-Induced AD Mice

**Figure 4:**
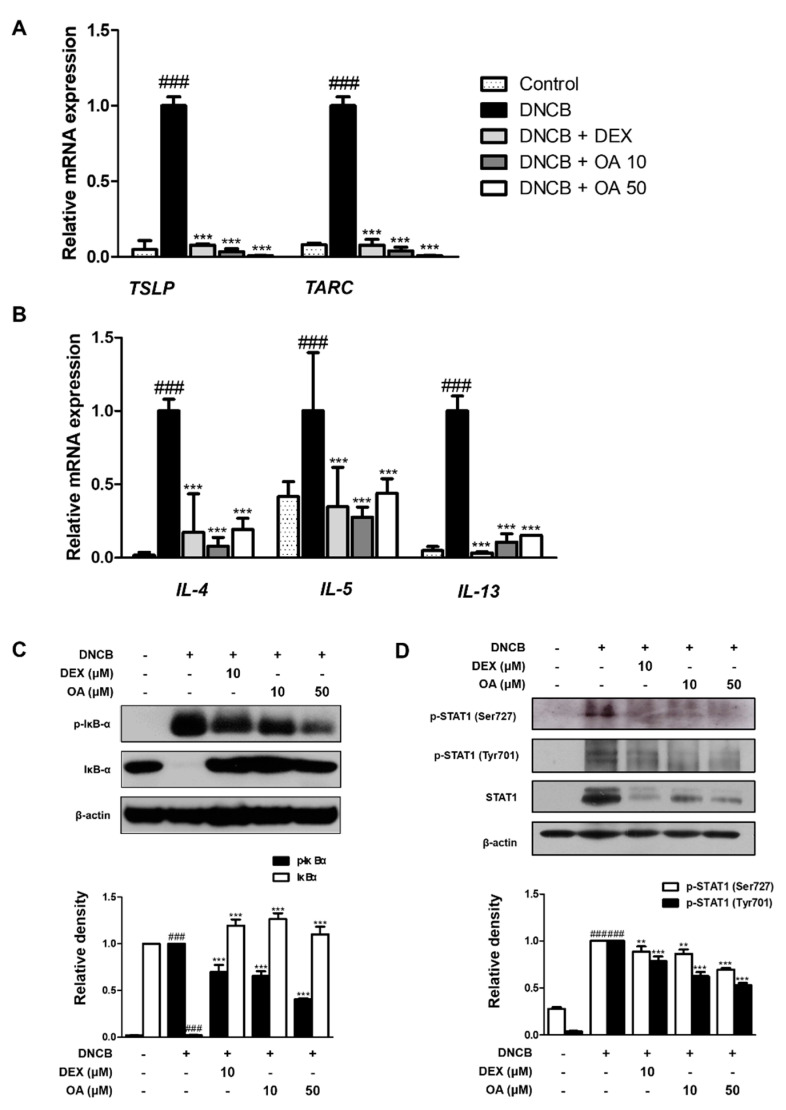
Effect of OA on AD cytokines and IκB, STAT1 activation in DNCB-induced AD skin lesions. Total RNA prepared from the dorsal tissue, and the level of (**A**) TARC, TSLP, (**B**) IL-4, IL-5, and IL-13 were determined by quantitative qRT-PCR. Expression of IκB (**C**) and STAT1 (**D**) was determined by Western blot analysis using specific antibodies. Densitometric analysis was performed using Bio-Rad Quantity One^®^ Software. The data shown represent mean ± S.D. (*n* = 6) of three independent experiments. ^###^ *p* < 0.001 vs. the control group; ** *p* < 0.01 and *** *p* < 0.001 vs. DNCB-treated group.

Next, we investigated whether OA inhibited the signature cytokines of AD in the dorsal tissues of DNCB-induced AD mice. Above all, thymus and activation-regulated chemokine (TARC)/CCL17, are members of the CC chemokine subfamily and are involved in the recruitment of Th2 lymphocytes and the continuation of Th2 immune responses [25]. In addition, thymic stromal lymphopoietin (TSLP) is known to provoke dendritic cell-mediated Th2 responses and is highly expressed in activated mast cells and skin of AD, which triggers allergic inflammation. Therefore, these cytokines are considered mediators of inflammatory skin diseases, such as AD [5,6,7,8,9,10,11,12,13,14,15,16,17,18,19,20,21,22,23,24,25,26]. As shown in Figure 4A, the mRNA expression levels of TSLP and TARC were markedly (*p* < 0.001) increased by repetitive treatment with DNCB, while OA reduced the expression levels of TSLP and TARC by approximately basal levels (*p* < 0.001). In line with these results, Th2-type cytokines, including IL-4, IL-5, and IL-13, were downregulated by OA treatment compared to DNCB-induced AD mice (*p* < 0.001) (Figure 4B).

To investigate the signaling pathways involved in the inhibitory effect of OA on cytokine production, we examined the phosphorylation and degradation of IκB and activation of STAT1 in DNCB-induced AD mice. The results demonstrated that the phosphorylation and degradation of IκB induced by DNCB were significantly (*p* < 0.001) inhibited by treatment with OA (Figure 4C). In addition, OA inhibited the DNCB-induced phosphorylation of STAT1 at residues Ser727 and Tyr701 with significance (Figure 4D). Considering our results, it can be presumed that the NF-κB and STAT1 signaling pathways are involved in the inhibitory effect of OA on the cytokine profiles of DNCB-induced AD-like skin.

### 2.4. OA Suppressed the Expression of AD Cytokines and Activation of NF-κB and Akt in TNF-α/IFN-γ-Stimulated HaCaT Keratinocytes

**Figure 5:**
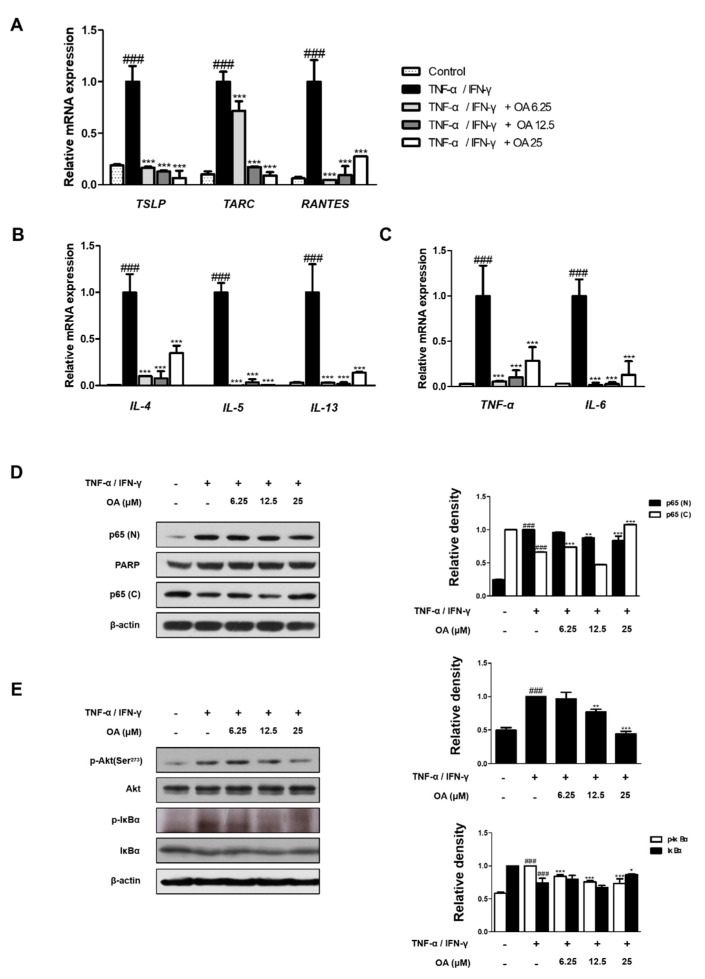
Effect of OA on chemokine and NF-κB and Akt activation in TNF-α/IFNγ-stimulated HaCaT cells (**A**–**C**) The mRNA level of cytokines and chemokines in HaCaT cells were determined by qRT-PCR. (**D**,**E**) Total proteins were prepared, and Western blot analysis was performed using specific antibodies. β-actin was used as internal control. Densitometric analysis was performed using Bio-Rad Quantity One^®^ Software. The data shown represent mean ± S.D. of three independent experiments. ^###^ *p* < 0.001 vs. the control group; * *p* < 0.05, ** *p* < 0.01, and *** *p* < 0.001 vs. TNF-α/IFN-γ-treated group.

To test in vivo findings in vitro, we investigated the effects of OA on pro-inflammatory cytokine expression in tumor necrosis factor (TNF)-α/IFN-γ-stimulated HaCaT keratinocytes. HaCaT human keratinocytes are the most commonly used cell line in the AD model, which produces various AD-related pro-inflammatory mediators in response to a variety of stimuli [27]. OA had no signs of toxicity up to 100 μM in HaCaT keratinocytes (Supplementary Figure S1). Many studies have shown that TSLP, TARC, and RANTES (Regulated on Activation, Normal T Cell Expressed and Secreted) play an active role in the pathogenesis of AD, and TNF-α/IFN-γ synergistically induces production of these mediators by human keratinocytes and HaCaT cells [28], and activation of NF-κB and STAT1 activation is involved in the production of chemokines and cytokines in keratinocytes [29]. Therefore, we investigated whether OA suppressed the production of AD cytokines and chemokines in TNF-α/IFN-γ-stimulated HaCaT cells by suppressing the NF-κB and STAT1 signaling pathways. First, we evaluated the mRNA levels of TSLP, TARC, and RANTES in TNF-α/IFN-γ-stimulated HaCaT cells. OA noticeably suppressed TNF-α/IFN-γ-stimulated mRNA expression of TSLP, TARC, and RANRES (*p* < 0.001) (Figure 5A), Th2 cytokines (*p* < 0.001) (Figure 5B), and pro-inflammatory cytokines, TNF-α and IL−6 (*p* < 0.001) (Figure 5C). These data suggest that OA can control the expression of Th2-related mediators and pro-inflammatory factors in TNF-α/IFN-γ-stimulated HaCaT cells. Next, we examined the effects of OA on the phosphorylation of NF-κB and Akt, which are signaling pathways that regulate inflammatory factors in human keratinocytes [30]. The results showed that OA reversed the nuclear translocation of NF-κB by treatment with 25 μM OA (*p* < 0.001), phosphorylation (*p* < 0.001), and degradation (*p* < 0.05) of IκB. Moreover, treatment with 25 μM OA prevented phosphorylation of Akt (*p* < 0.001), which promotes the activation of NF-κB by directly phosphorylating IκB kinase in response to stimuli, but it did not affect the total amount of Akt in TNF-α/IFN-γ-stimulated HaCaT cells (Figure 5D,E). These results suggest that OA might suppress TNF-α/IFN-γ-induced cytokines and chemokines by suppressing NF-κB and Akt in HaCaT keratinocytes.

### 2.5. OA Inhibited STAT1 Activation in TNF-α/IFN-γ-Stimulated HaCaT Keratinocytes

**Figure 6:**
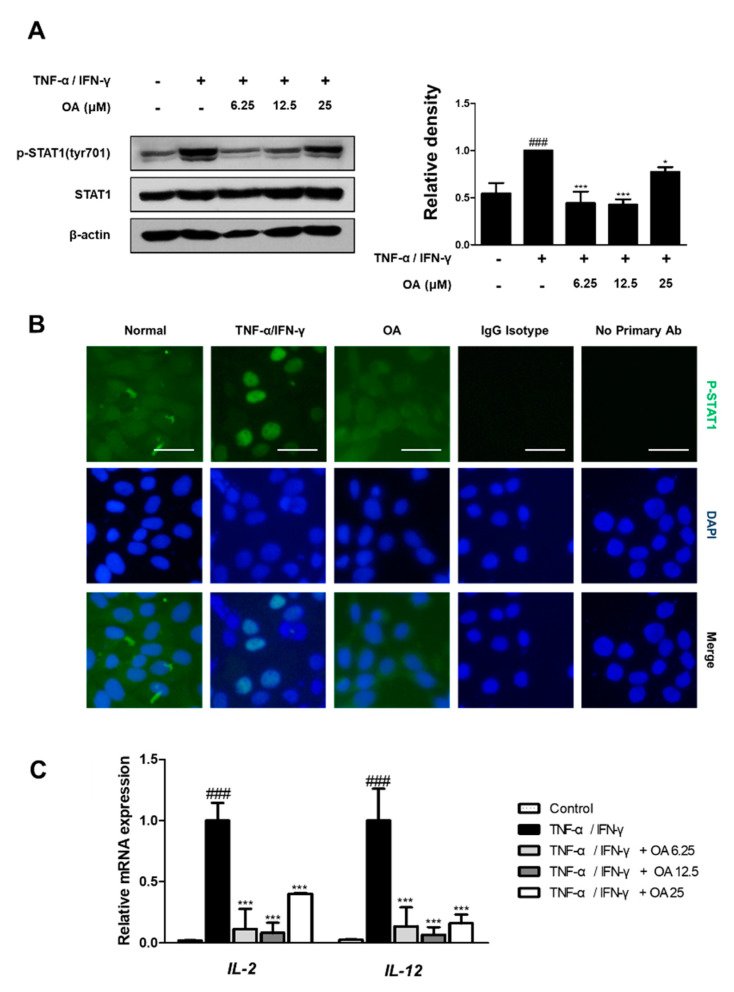
Effect of OA on STAT1 activation in TNF-α/IFNγ-stimulated HaCaT cells. (**A**) Total proteins were prepared, and Western blot analysis was performed using specific antibodies. β-actin was used as internal control. Densitometric analysis was performed using Bio-Rad Quantity One^®^ Software. (**B**) Immunofluorescence staining of p-STAT1 (green) in TNF-α/IFN-γ-stimulated HaCaT keratinocytes. Nuclei were counterstained using DAPI. IgG isotype control and secondary antibody only control were used as negative control (scale bar = 100 μm). (**C**) The mRNA level of IL-2, IL-12, and IL-8 in HaCaT cells were determined by qRT-PCR. The data shown represent mean ± S.D. of three independent experiments. ^###^ *p* < 0.001 vs. the control group; * *p* < 0.05, and *** *p* < 0.001 vs. TNF-α/IFN-γ-treated group.

To investigate the mechanism responsible for the inhibitory effect of OA in parallel with the inhibition of NF-κB, we investigated its effect on TNF-α/IFN-γ-induced STAT1 activation. As shown in Figure 6A,B, treatment with TNF-α/IFN-γ resulted in the activation of STAT1 (*p* < 0.001). However, OA decreased the phosphorylation at Tyr701 with significance and alleviated the nuclear translocation of STAT1 in TNF-α/IFN-γ-stimulated HaCaT cells (Figure 6B). In addition, the expression of cytokines, such as IL-2 and IL-12, which are involved in the phosphorylation of STAT1, was downregulated by OA treatment compared to TNF-α/IFN-γ-treated HaCaT cells (*p* < 0.001) (Figure 6C). The results demonstrated that the anti-inflammatory effects of OA in HaCaT and DNCB-induced AD mice are associated with the STAT1 signaling pathway.

## 3. Discussion

### 3.1. AD Phenotypes Reflected in This Study

AD is a complex disease, whose relationship with allergy remains controversial and can be viewed from different perspectives. AD is recognized as a type I and type IV complex according to the Coombs and Gell classification system [31]. Epidermal keratinocytes provide a functional skin barrier on the frontline of the defense system. In the skin of patients with AD, allergic sensitization, due to pathogen invasion, irritants, and allergens, occurs through a damaged skin barrier that leads to the stimulation of immune responses in keratinocytes, with increased skin inflammation [23,24,25,26,27,28,29,30,31,32]. The expression of various inflammatory cytokines and chemokines derived from keratinocytes plays important roles in the pathogenesis of AD [33,34]. Although a biphasic immune response is observed, a Th2-biased immune response (IL-4, IL-13, TSLP, and eosinophils) is predominant in the initial and acute phases of AD [35].

Based on the well-known anti-allergic and anti-inflammatory properties of OA, we adopted the DNCB-induced AD model to evaluate the effect of OA on AD mice in vivo. DNCB induces contact dermatitis accompanying skin and immunological alterations in mice, which is similar to aspects observed in human patients with AD, including clinical features, such as skin dryness, erosion, edema, hemorrhage, erythema, and increased serum IgE levels [2]. In this study, we confirmed that OA alleviated the symptoms in patients with AD, such as hypertrophy of the epidermal layer (Figure 1 and Figure 2), recruitment of inflammatory cells, and histamine release (Figure 3), using histological methods. Since AD is thought to be a Th2-dominant inflammatory skin disease in the acute phase, TARC is thought to be implicated in the pathogenesis of AD [36], correlating with the severity of disease in some chronic allergic pathologies, such as asthma and AD [37]. TSLP is an epithelial cell-derived cytokine [38] and is strongly implicated in the pathogenesis of Th2 cell-mediated allergic disorders. Eventually, TSLP specifically increased the frequency of IL-4-, IL-5-, and IL-13-expressing effector cells, which enhanced cytokine production [39]. In this regard, we investigated the effect of OA on the mRNA expression of TSLP and TARC. The results showed that OA significantly decreased the mRNA levels of TSLP and TARC in the DNCB-induced AD animal model (Figure 4A). Furthermore, OA inhibited the DNCB-induced phosphorylation and degradation of IκB and the phosphorylation of STAT1 at both residues. These results indicate the possibility that the promoter of TSLP or TARC includes NF-κB- and STAT-binding sequences, so that these transcription factors may regulate the transcription of TSLP and TARC, as reported previously [40,41].

### 3.2. The Mechanisms of OA in This Study

These results prompted us to confirm how OA regulates AD development. Therefore, we investigated the mechanism of action of OA in AD using human-derived keratinocytes. OA suppressed the mRNA levels of TSLP, TARC, RANTES, and Th1/Th2 type cytokines in TNF-α/IFN-γ-stimulated HaCaT keratinocytes. Moreover, the TNF-α/IFN-γ-induced phosphorylation and degradation of IκB, translocation of NF-κB, and phosphorylation of Akt and STAT1 (Tyr701) were inhibited by pretreatment with OA. These results indicate that the regulation of cytokine and chemokine expression in HaCaT keratinocytes is related to the Akt, NF-κB, and STAT1 signaling pathways. Previous studies have shown that the mitogen-activated protein kinase (MAPK) signaling pathway is involved in the regulation of NF-κB and STAT1 in response to cytokines, such as TNF-α and IFN-γ [42,43,44]. However, the results indicated that OA did not suppress TNF-α/IFN-γ-induced phosphorylation of MAPKs including ERK, JNK/SAPK, and p38 MAPK (data not shown), suggesting that the expression of cytokines and chemokines in TNF-α/IFN-γ-stimulated HaCaT keratinocytes via suppression of other pathways, such as other STAT family members under the action of OA, judging from the multiple roles of cytokines. However, it is necessary to examine how OA exerts its action via a certain receptor at the upstream signaling pathway. Previous reports have described that anti-inflammatory activities of OA were related to inhibition of toll-like receptors (TLRs) signaling pathway and consequent inflammatory responses [45,46]. TLRs induce the MyD88-dependent pathway to mediate downstream inflammatory pathways through activation of NF-κB [47], supporting our results regarding downregulation of the NF-κB pathway. Moreover, engagement of IFN-γ receptor signaling, which leads to STAT1 activation and the receptors of various cytokines also should be considered to clarify the roles of OA in AD pathology. Despite these limitations, in this study, we provided potential mechanisms through which OA exerts its role in allergic inflammation. The activated Akt, NF-κB, and STAT1 pathways mediated the signal transduction triggered by allergen, and the mediators induced the development AD symptoms, including skin inflammatory response, immune cell infiltration, and Th2 cytokines production. However, OA alleviates AD symptoms through the downregulation of Akt, NF-κB, STAT1 signaling pathways and the expression of AD-associated cytokines, suggesting the potential therapeutic targets.

### 3.3. For Improving the Activity of OA in Skin

We conducted the present study to evaluate the anti-AD effects of OA in in vivo and in vitro models. Consistent with a previous study that found that oral administration of oleanolic acid acetate, the derivative of oleanolic acid, suppressed DNCB-induced atopic skin symptoms in BALB/c animal models [48], we confirmed that OA alleviated atopic symptoms, despite the difference in mouse strains. Our study showed that OA decreased the serum histamine levels which is induced by DNCB (Figure 3C), indicating that the topical application of OA can affect the systemic immune system as well as local skin barrier. Due to its low water solubility, there have been several attempts to improve the water solubility and bioavailability of OA such as polymeric micelles [49], nanoemulsions [50], nanoliposomes [51] containing oleanolic acid. Studies on routes of administration of OA including topical medication are required for preclinical efficacy. It is expected that developed dosage forms of OA can be widely used in skin disorders. Furthermore, it is necessary to clarify the upstream event of the identified molecular mechanisms of OA, along with its effect on skin barrier-related markers. Further studies based on immune responses, such as the effect of OA on toll-like receptors, innate lymphoid cells, and T cell responses, are needed to understand the anti-allergic inflammatory effect of OA and develop a therapeutic approach to treat allergic disorders.

In conclusion, OA inhibited AD-like responses, suppressing the pathway to make up the atopic environments dominated by Th2 cells via the inhibition of cytokines derived from skin keratinocytes via the blockade of Akt, NF-κB, and STAT1 signaling pathways. Considering this, we suggest that OA can be used as a potential therapeutic agent for the prevention or treatment of allergic inflammatory diseases, such as AD. We believe that these findings can be further elucidated in future studies to determine the specific roles of OA.

## 4. Materials and Methods

### 4.1. Chemicals and Reagents

For the present study, OA (O5504, ≥97%), 3-(4,5-Dimethylthiazol-2-yl)-2,5-diphenyl tetrazolium bromide (MTT, >98%), dimethyl sulfoxide (DMSO, ≥99.9%), and all other chemicals were purchased from Sigma; EMD Millipore (Billerica, MA, USA). Recombinant human TNF-α and recombinant human IFN-γ were purchased from Bio-Techne Ltd. (Abingdon, OX, UK). Dulbecco’s modified Eagle medium (DMEM), fetal bovine serum (FBS), penicillin, and streptomycin were obtained from Life Technologies Inc. (Grand Island, NY, USA). Primary antibodies against NF-κB p65 (cat no. 8242), p-Akt (cat no. 9271), p-STAT1 (Tyr701; cat no. 9167), and p-STAT1 (Ser727; cat no. 8826), STAT1 (cat no. sc-14994), and IgG XP^®^ Isotype (cat no. 3900) were obtained from Cell Signaling Technology, Inc. (Danvers, MA, USA). Primary antibodies for p-IκB-α (cat no. sc-8404), IκB-α (cat no. sc-203), Akt1/2/3 (cat no. sc-8312), PARP (cat no. sc-9542), α-tubulin (cat no. sc-8035), and β-actin (cat no. sc-81178) were purchased from Santa Cruz Biotechnology, Inc. (Dallas, TX, USA). Horseradish peroxidase-conjugated secondary antibodies were purchased from Jackson ImmunoResearch laboratories, Inc. (West Grove, PA, USA). The histamine enzyme-linked immunosorbent assay (ELISA) kit was obtained from Enzo life Sciences, Inc. (Farmingdale, NY, USA). SYBR Premix Ex Taq was purchased from Takara Bio, Inc. (Kusatsu, Japan). Oligonucleotide primers were purchased from Bioneer Corporation (Daejeon, Korea).

### 4.2. DNCB-Induced AD Model

A total of 30 ICR female mice (6 weeks old; 20–25 g body weight) were obtained from Charles River Laboratories (Harlan laboratories, inc., Wilmington, MA, USA) and maintained under constant conditions at a humidity of 40–60%, temperature of 20–25 °C, and a 12 h light/dark cycle. The mice were randomly assigned to one of five groups (*n* = 6 per group). To induce AD-like symptoms and skin lesions, 1-Chloro-2,4-dinitrochlorobenzene (DNCB, 97%) was used. Briefly, the mice were sensitized topically with 100 μL of 1% DNCB dissolved in 4:1 *v*/*v* mixture of acetone and corn oil and topically applied on the shaved area of the dorsal surface of mice for three days, followed by no treatment for four days. The same volume of vehicle (acetone/corn oil) was applied to the Normal group. After the first challenge inducing the AD-like symptoms, the treatment was repeated with 100 μL of 0.5% DNCB for 21 days. The mice were topically applied vehicle, dexamethasone (≥97%, 10 μM), or OA (10 and 50 μM) 4 h after DNCB treatment once a day. Dexamethasone is dissolved in PBS:100% EtOH:Cremophor (6:1:3). OA is dissolved in 100% EtOH:Cremophor (7:3). Mice were sacrificed on day 29 of the experiment. The experimental scheme is summarized in Figure 1A. Skin tissues from the back of the mice were obtained and subjected to histological analysis, qRT-PCR examination. All procedures were performed in accordance with university guidelines and approved by the Instructional Animal Care and Use Committee (IACUC) of Korean Medicine, Sangji University (Wonju, Korea; approval no. 2015-06).

### 4.3. Evaluation of Dermatitis Severity

Clinical dermatitis severity was tested using the method described by Yamamoto and colleagues (35). The severity of dermatitis was evaluated at the experiment start day and end day. The development of erythema/hemorrhage, scarring/dryness, edema, and excoriation/erosion was scored as follows: 0, none; 1, mild (<20%); 2, moderate (20–60%); 3, severe (>60%). The scores were determined in agreement between three observers, and the sum of the individual scores was used as the dermatitis score.

### 4.4. Histopathological Analysis

At the end of the study period, the dorsal skin of mice was collected. The samples were fixed in 10% buffered formalin, embedded in paraffin, sectioned into 4 μm thick, and stained with hematoxylin and eosin (H&E) and toluidine blue to detect epidermal thickness and inflammatory cells. Pathological changes of all stained skin sections were observed using a DM IL LED microscope (Leica, Wetzlar, Germany) and photographed using a DFC295 (Leica, Wetzlar, Germany). Digital images were taken from each slide (2 per group), and measured using Leica Application Suite (Leica, Wetzlar, Germany).

### 4.5. Histamine Assay

Blood from the mice was collected from each mouse at the end of the experiment. Serum was obtained by centrifugation at 1700× *g* for 30 min and stored at −80 °C until analysis. The release of histamine was measured using an ELISA kit in accordance with the manufacturer’s protocol.

### 4.6. Cell Culture and Sample Treatment

HaCaT keratinocytes (passage 19) were provided by Professor Jae-Young Um (Kyung Hee University, Seoul, Korea), and were grown at 37 °C in DMEM supplemented with 10% FBS, penicillin (100 U/mL) and streptomycin (100 μg/mL) in a humidified atmosphere of 5% CO_2_. HaCaT keratinocytes were seeded at a density of 1 × 10^5^ cell per well, starved with 0.1% FBS media for 24 h, and treated with OA at 6.25, 12.5, and 25 μM for 1 h at 37 °C in humidified air with 5% CO_2_, and then stimulated with 10 ng/mL of TNF-α/IFN-γ at 37 °C for indicated time. The cells were either treated with DMSO as a control. The OA was dissolved in DMSO.

### 4.7. Western Blot Analysis

Segments of cells, or dorsal tissue were suspended in PRO-PREP™ protein extraction solution (Intron Biotechnology, Inc., Seoul, Korea) and incubated for 20 min at 4 °C. Cell debris was removed via micro-centrifugation 11,000× *g* for 30 min at 4 °C, followed by rapid freezing of the supernatant. The protein concentration was determined using Bio-Rad protein assay reagent (Bio-Rad Laboratories, Inc., Hercules, CA, USA) according to the manufacturer’s protocol. Cellular proteins from the treated and untreated cell extracts (10–30 μL) were electroblotted onto a polyvinylidene fluoride membrane following separation via 8–12% SDS-PAGE. The membrane was incubated for 1 h with blocking solution (5% skim milk) at room temperature, followed by overnight incubation with the primary antibodies (1:1000) at 4 °C. The blots were washed three times with Tween 20/Tris-buffered saline (T/TBS) and incubated with horseradish peroxidase-conjugated secondary antibody (1:2000) for 2 h at room temperature. The blots were washed three times with T/TBS, and then developed via enhanced chemiluminescence (GE Healthcare Life Sciences, Chalfont Saint Giles, UK). Densitometric analysis was performed using Bio-Rad Quantity One software version 4.3.0 (Bio-Rad Laboratories, Inc., Hercules, CA, USA).

### 4.8. Reverse Transcription-Quantitative Polymerase Chain Reaction (RT-qPCR) Analysis

Total RNA was isolated from the cells, or dorsal tissue using an Easy Blue kit (Intron Biotechnology, Inc., Seoul, Korea) according to the manufacturer’s protocol. Total RNA was quantified using an Epoch micro-volume spectrophotometer system (BioTek Instruments, Inc., Winooski, VT, USA). cDNA was obtained using isolated total RNA (2 μg), d (T)16 primer, and Avian Myeloblastosis Virus reverse transcriptase with genomic DNA remover. The relative gene expression was quantified using RT-qPCR analysis (Real Time PCR System 7500; Applied Biosystems; Thermo Fisher Scientific, Inc., Waltham, MA, USA) with SYBR Premix Ex Taq. Fold changes of gene expression were calculated using the comparative quantification cycle (Cq) method. The Cq values of target genes were normalized to that of GAPDH using the ABI gene express 2.0 program (Applied Biosystems; Thermo Fisher Scientific, Inc., Waltham, MA, USA).

### 4.9. Immunofluorescence Staining

Cells were seeded in a chamber at 1 × 10^5^ cells/mL, fixed with 100% methanol for 30 min at 20 °C, and blocked in 10% NGS in 0.3% Triton-X100 (Sigma). The samples were then incubated with p-STAT1 primary antibody or IgG isotype control overnight at 4 °C. After washing, the samples were incubated with the secondary antibody with Alexa-Fluor 488-conjugated goat anti-rabbit IgG (Invitrogen, Waltham, MA, USA). Coverslips were mounted on to the glass slides and the images were captured on a confocal laser-scanning fluorescence microscope Leica TCS SP5 (LAS AF) suite (Leica Microsystems, Wetzlar, Germany).

### 4.10. Statistical Analysis

The data are expressed as the mean ± standard deviation of triplicate experiments. Statistically significant differences were compared using one-way analysis of variance (ANOVA) with Dunnett’s post hoc test. *p* < 0.05 was considered to indicate a statistically significant difference. Statistical analysis was performed using GraphPad Prism (version 5).

## Data Availability

The data presented in this study are available on request from the corresponding author.

## Supplementary Materials

The following are available online at https://www.mdpi.com/article/10.3390/ijms222112000/s1.
