# Supplementary material for: Oleanolic Acid Alleviates Atopic Dermatitis-like Responses In Vivo and In Vitro
Source: Int J Mol Sci. 2021 Nov 5;22(21):12000. doi: 10.3390/ijms222112000 (PMC8584529; doi:10.3390/ijms222112000)
Supplement: Supplementary file 1 [file ijms-22-12000-s001.zip › ijms-1431016-supplementary.pdf]

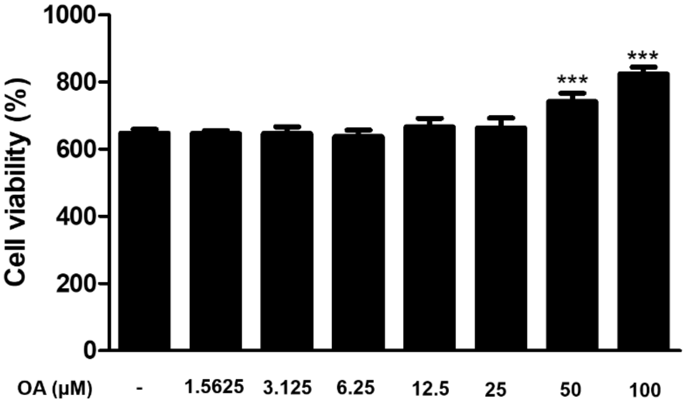

Supplementary Figure S1. Effect of OA on cell viability in HaCaT cells. Cells were seeded in a 96-well culture plate at 5×10^4 cells/ml in culture medium and allowed to attach for 24 h. Cells were treated with medium containing various concentrations of OA. After incubating for 24 h, the cells were treated with 50 µl of MTT (5 µg/ml) for 4 h. The formazan precipitated was dissolved in DMSO, and absorbance was measured at 540 nm using a microplate reader.
